# X-ray in-line holography and holotomography at the NanoMAX beamline

**DOI:** 10.1107/S1600577521012200

**Published:** 2022-01-01

**Authors:** Sebastian Kalbfleisch, Yuhe Zhang, Maik Kahnt, Khachiwan Buakor, Max Langer, Till Dreier, Hanna Dierks, Philip Stjärneblad, Emanuel Larsson, Korneliya Gordeyeva, Lert Chayanun, Daniel Söderberg, Jesper Wallentin, Martin Bech, Pablo Villanueva-Perez

**Affiliations:** a MAX IV Laboratory, Lund University, 22100 Lund, Sweden; b Division of Synchrotron Radiation Research and NanoLund, Department of Physics, Lund University, 22100 Lund, Sweden; c Univ. Grenoble Alpes, CNRS, UMR 5525, VetAgro Sup, Grenoble INP, TIMC, 38000 Grenoble, France; d Department for Medical Radiation Physics, Clinical Sciences Lund, Lund University, 221 85 Lund, Sweden; e Excillum AB, Jan Stenbecks Torg 17, 16440 Kista, Sweden; f Division of Solid Mechanics and LUNARC, Department of Construction Sciences, Lund University, 22100 Lund, Sweden; g Wallenberg Wood Science Center, Department of Fibre and Polymer Technology, KTH Royal Institute of Technology, 10044 Stockholm, Sweden

**Keywords:** holography, holotomography, 2D and 3D X-ray imaging, coherent imaging, diffraction-limited storage ring

## Abstract

Coherent X-ray imaging techniques, such as in-line holography, exploit the high brilliance provided by diffraction-limited storage rings to perform imaging sensitive to the electron density through contrast due to the phase shift, rather than conventional attenuation contrast. Thus, coherent X-ray imaging techniques enable high-sensitivity and low-dose imaging, especially for low-atomic-number (*Z*) chemical elements and materials with similar attenuation contrast. Here, the first implementation of in-line holography at the NanoMAX beamline is presented, which benefits from the exceptional focusing capabilities and the high brilliance provided by MAX IV, the first operational diffraction-limited storage ring up to approximately 300 eV. It is demonstrated that in-line holography at NanoMAX can provide 2D diffraction-limited images, where the achievable resolution is only limited by the 70 nm focal spot at 13 keV X-ray energy. Also, the 3D capabilities of this instrument are demonstrated by performing holotomography on a chalk sample at a mesoscale resolution of around 155 nm. It is foreseen that in-line holography will broaden the spectra of capabilities of MAX IV by providing fast 2D and 3D electron density images from mesoscale down to nanoscale resolution.

## Abstract 2

First results of in-line holography and holotomography from the NanoMAX beamline at MAX IV are presented.

## 1. Introduction

Diffraction-limited storage rings offer an unprecedented brilliance, which opens an opportunity for new science (Eriksson *et al.*, 2014 ▸). High brilliance or coherent flux is instrumental in increasing (i) the portion of flux that can be focused into a diffraction-limited spot, and (ii) the capabilities of coherent X-ray imaging techniques (Mayo *et al.*, 2002 ▸; Chapman & Nugent, 2010 ▸; Pfeiffer, 2018 ▸). Among current coherent X-ray imaging techniques, in-line holography (Gabor, 1948 ▸) benefits from both improvements provided by highly brilliant sources. The resolution of in-line holography in magnified geometries is limited by the focal-spot size (Mayo *et al.*, 2002 ▸; Mokso *et al.*, 2007 ▸; Kalbfleisch *et al.*, 2010 ▸; da Silva *et al.*, 2017 ▸). Thus, smaller focal spots with higher flux will enable higher resolutions and faster acquisition.

MAX IV (Tavares *et al.*, 2014 ▸) is the first operational diffraction-limited storage ring. Its horizontal emittance of 326 pm rad provides a diffraction-limited source up to 306 eV (Johansson *et al.*, 2021 ▸). Among the beamlines that exploit the unique brilliance of MAX IV, NanoMAX (Johansson *et al.*, 2021 ▸) is a hard X-ray beamline with focusing capabilities down to the nanometre scale. Due to its focusing capabilities and high coherent flux, NanoMAX is used for a combination of scanning techniques, such as X-ray fluorescence (Arai *et al.*, 2006 ▸; Silva Barreto *et al.*, 2020 ▸), X-ray diffraction (Warren, 1969 ▸; Björling *et al.*, 2019 ▸), and scattering techniques (Birkholz *et al.*, 2005 ▸; Nissilä *et al.*, 2021 ▸). Other techniques implemented at NanoMAX that exploit its unique capabilities are coherent X-ray imaging techniques, such as ptychography (Pfeiffer, 2018 ▸) and coherent diffraction imaging (Chapman & Nugent, 2010 ▸). These techniques are routinely used and are under continuous development. The first 3D ptychographic reconstruction from NanoMAX has been recently reported (Kahnt *et al.*, 2020 ▸), clearly demonstrating the 3D nanoscopic capabilities of NanoMAX.

Here, we present the first implementation of in-line holography at NanoMAX. We demonstrate the capabilities of this instrument to bridge the resolutions and imaging capabilities of micro-computed tomography (µCT) (Landis & Keane, 2010 ▸) and nano-computed tomography (nanoCT) by exploiting the unique focus and coherent flux provided by NanoMAX. To demonstrate this, we retrieved 2D diffraction-limited images from test samples. Furthermore, we performed tomographic reconstructions of a chalk sample, retrieving resolutions at around 155 nm.

The remainder of this paper is structured as follows: First, we describe and present the current features of the in-line holography instrument at NanoMAX. Second, we present the 2D and 3D results and analyze the resolutions obtained. Finally, we discuss the obtained results and present ideas to further optimize the setup to fully exploit the unique capabilities of NanoMAX to explore the mesoscale.

## 2. Experiment

### 2.1. Experimental setup

**Figure 1:**
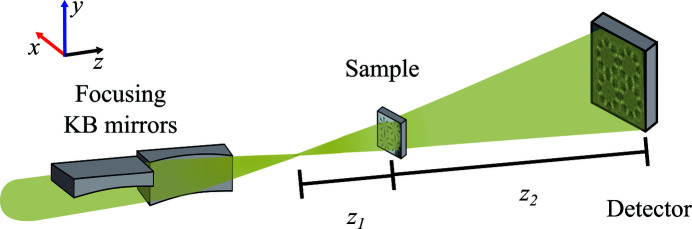
In-line holography. Conceptual sketch of in-line holography at the NanoMAX beamline.

This experiment established an in-line holography microscope at the NanoMAX beamline. A conceptual illustration of the setup can be found in Fig. 1 ▸.

The in-line holography setup at NanoMAX exploits the nanofocus provided by a pair of orthogonal Kirkpatrick–Baez (KB) mirrors (Johansson *et al.*, 2013 ▸, 2018 ▸; Osterhoff *et al.*, 2019 ▸), and the focal spot size ultimately determines the resolution (Mokso *et al.*, 2007 ▸). The KB system at NanoMAX is aperture-limited rather than limited by the critical angle. As a result, the focal spot size depends on the incoming X-ray photon energy. The focal spot and wavefront as a function of the incoming photon energy have been characterized using ptychography (Vila-Comamala *et al.*, 2011 ▸; Björling *et al.*, 2020 ▸). For example, for 6, 14, and 22 keV X-ray photons, the KB system can obtain focal spot sizes of 151, 64, and 42 nm, respectively (Björling *et al.*, 2020 ▸). Thus, by selecting different energies, we can explore different resolutions with in-line holography.

Samples were mounted on the NanoMAX diffractometer stage (Johansson *et al.*, 2013 ▸). The diffractometer stage allowed for the angular alignment of a precise and long-range travel motor parallel to the optical axis. By aligning the motorized axis to the optical axis, we could keep a region of interest within the sample illuminated while modifying the sample-to-focus distance, *i.e.* effectively changing the magnification of the microscope. The current setup has a travel range of 4 cm, which allows positioning the sample in focus and moving it up to approximately 4 cm downstream of the focus (*z*_1_ = 4 cm). This range of motion enables the combination of in-line holography with other scanning techniques such as X-ray fluorescence. However, our motorized travel range was limited to 2 cm during these experiments. Two extra pairs of motors for coarse and fine positioning were used to position and scan the sample in the *x* and *y* directions, using the axes convention depicted in Fig. 1 ▸. On top of the scanning and positioning motors, a small and light rotation stage (XERYON XRT-A-25-109) was mounted. This stage was previously commissioned for ptychographic X-ray computed tomography (Kahnt *et al.*, 2020 ▸). A manual 2D alignment stage was mounted on top of the rotation stage to position the sample in the center of rotation. For such alignment, the NanoMAX optical on-axis microscope was used.

As an X-ray detector, we used a CRYCAM microsystem from Crytur. This indirect-detection system uses: a LuAG(Ce) scintillator to convert X-ray photons to visible light, 10× visible optics, and an Andor Zyla 4.2 Plus Camera. The effective pixel size of this detection system is 650 nm with an effective field-of-view (FOV) of 1.3 mm × 1.3 mm. The detector was positioned at 1.12 m from the focal spot (*z*_1_ + *z*_2_) to illuminate the whole sensor, given the divergence of the KB system of 2θ ≃ 1.2 mrad.

### 2.2. Measurements

To demonstrate the capabilities of the in-line holography instrument at NanoMAX, we measured a test pattern in 2D and a sample in 3D. The 2D test pattern was fabricated at the NanoLund laboratory, and consists of a stack of Pd/Zn/Pd/Au metal layers with thicknesses of 21/10/11/163 nm, respectively, deposited on a 1 µm-thick silicon nitride membrane (Chayanun *et al.*, 2018 ▸).

The 3D chalk sample was obtained after smashing a chalk piece and selecting a grain with a diameter below 20 µm. As expected from electron microscopy images, this sample has features ranging from the microscale up to the nanoscale.

To resolve the features relevant for our samples, we decided to perform our experiments at 13 keV X-ray photon energy. At this photon energy, the KB mirrors provide a diffraction-limited focal spot of approximately 70 nm (FWHM) (Björling *et al.*, 2020 ▸). The focal spot size and position were estimated using forward X-ray ptychography on 2D test patterns (Björling *et al.*, 2020 ▸; Kahnt *et al.*, 2020 ▸).

**Figure 2:**
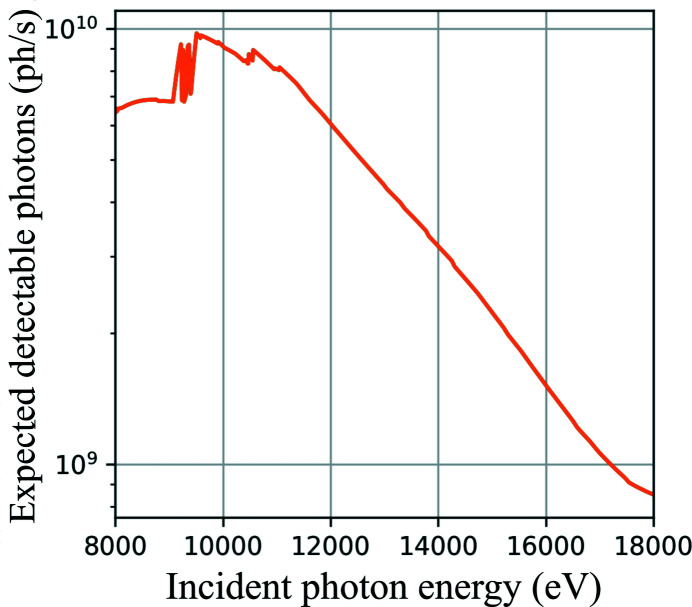
Expected detectable photons with in-line holography at NanoMAX as a function of the X-ray photon energy.

Since the resolution in in-line holography is limited by the focal spot size, it is also energy-dependent for the used KB system. Choosing a higher photon energy would reduce the focal spot size and interaction with air but also results in a lower detector efficiency and flux of NanoMAX. Therefore, 13 keV was chosen as a compromise between detector efficiency, available coherent flux, and diffraction-limited X-ray beam focus size, thus, achievable resolution (see Fig. 2 ▸).

Figure 2 ▸ represents the expected detectable photons per unit of time of this setup as a function of the X-ray photon energy. This figure of merit is calculated by multiplying the known available coherent flux (Björling *et al.*, 2020 ▸) with the transmissivity of 1 m of air (as used in the presented experiment) and the absorption of the 10 µm-thick scintillator.

**Figure 3:**
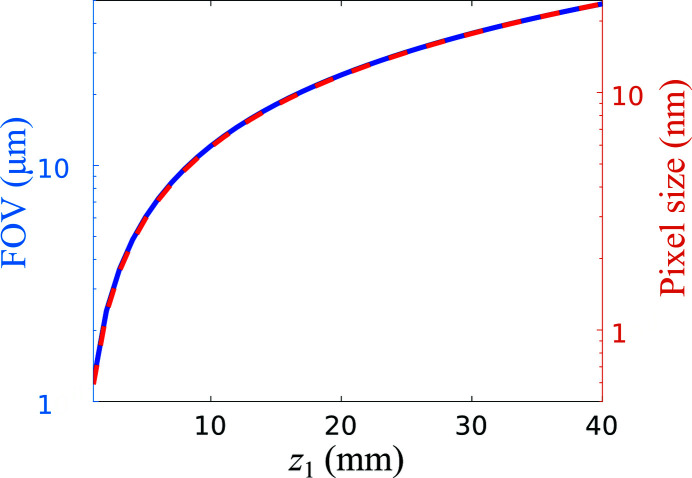
Field of view (FOV) and effective pixel size as a function of the defocusing distance (*z*_1_) for the NanoMAX in-line holography setup.

To reach a diffraction-limited resolution, we also need the ability to sample at the target resolution. This is achieved by exploiting the small pixel size of the CRYCAM microsystem together with the geometrical magnification provided by the KB system. As aforementioned, the total length of the setup is determined by the CRYCAM effective sensor size and the divergence of the KB system. These constraints determine the magnification or maximum focus-to-sample distance (*z*_1_), thus enabling diffraction-limited resolutions. Figure 3 ▸ depicts the approximate FOV and effective or demagnified pixel size as a function of *z*_1_ for the CRYCAM microsystem when optimized for the NanoMAX KB system. The 2D test sample was scanned at *z*_1_ = 10.1, 15.7, 18.0, 19.3, and 20.6 mm with an acquisition time of 1 s per frame. At these positions, the transverse coherence given by the 70 nm secondary source at 13 keV was 13.8, 21.4, 24.5, 26.3, and 28.1 µm. Given the divergence of the KB system, these quantities are larger than the illumination at those positions. The *z*_1_ positions were estimated using an optical on-axis microscope.

To perform such an estimation, we recorded a reference position by simultaneously positioning the sample on the focus of the on-axis optical microscope and the X-ray beam, as estimated by ptychography. When the sample is out-of-focus with respect to the X-ray focus position, the displacement between the sample and the X-ray focus position (*z*_1_) was measured by moving the optical microscope along the optical axis until the sample was in the optical-microscope focus.

**Figure 4:**
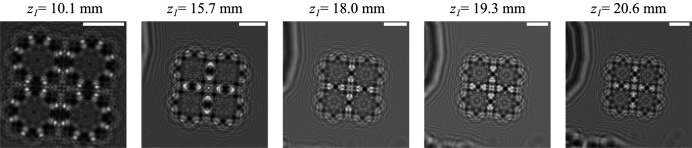
Recorded holograms at different defocusing distances (*z*_1_) of the 2D test pattern. The white scale bar corresponds to 4 µm for all the images.

These positions were selected to optimize the phase reconstruction by combining them in the phase reconstruction step (Zabler *et al.*, 2005 ▸). In order to estimate the effective or demagnified pixel size, a 2D mesh scan was performed. By performing registration on a known translation, we could estimate the pixel size on each plane. The estimated pixel sizes for the 2D test sample were 6.0, 9.4, 10.7, 11.5, and 12.3 nm from the increasing value of *z*_1_, while keeping the focus-to-detector distance constant (*z*_1_ + *z*_2_ = 1.12 m). Figure 4 ▸ depicts the recorded holograms at the different defocusing distances for the 2D test pattern.

Finally, we performed holographic nanotomography on the chalk sample at 13 keV. We acquired 1300 projections from 0 to 180° at four different defocusing distances (*z*_1_ = 28.0, 31.4, 34.9, and 38.4 mm) with an acquisition time per projection of 1 s. The number of projections is selected as the double quantity required by the Crowther criterion. The Crowther criterion is calculated assuming a pixel size of 15 nm, a desired resolution of 100 nm, and a region of interest contained in a cylinder with a diameter of fewer than 1380 pixels. The effective pixel sizes corresponding to those focus-to-sample distances were 15.0, 17.3, 19.2, and 21.2 nm, respectively. These pixel sizes resulted in FOVs of 30.7, 35.4, 39.3, and 43.4 µm for the CRYCAM microsystem.

## 3. Results

### 3.1. Reconstruction procedure

This subsection describes the procedure to retrieve 2D and 3D phase reconstructions using in-line holography at NanoMAX.

First, we performed flat-field corrections to the acquired data. For such purpose, we recorded: (i) flat frames, *i.e.* images without the sample, and (ii) dark frames, *i.e.* images without X-rays that estimate the electronic noise of the acquisition system. Flat and dark frames were collected every time we performed an acquisition at a different defocusing distance. Averaged flat and dark images were used together with the data to obtain flat-field corrected images to reduce the fixed-pattern noise.

Second, we performed image registrations for the different flat-field-corrected images recorded at different defocusing distances in order to prepare them for phase reconstruction using these multiple defocusing distances. This process comprised two steps. The first one was demagnification, *i.e.* we used the physical size of the effective pixel to bring all the images to the same scale. In our case, we brought all the images to the largest magnification. The second step was a similarity registration that includes rotation, translation, and scaling operations. The scaling was introduced to refine the estimated demagnifications. Given that the propagation artifacts are different for each defocusing distance, performing a simple intensity registration approach was not possible. To address this issue, we used mutual information methods (Weber *et al.*, 2018 ▸). The described process is implemented in *PyPhase* (Langer *et al.*, 2021 ▸), a modular open-source phase-reconstruction package implemented in Python. Specifically for the registration, we used *Elastix* (Klein *et al.*, 2010 ▸; Shamonin *et al.*, 2014 ▸) via its Python wrapper *PyElastix* (Klein, 2019 ▸).

Third, we performed phase reconstructions for each individual projection by combining the registered images at different defocusing distances. The phase reconstructions were performed using contrast-transfer function approaches (CTFs) (Guigay, 1977 ▸) implemented in *PyPhase* (Langer *et al.*, 2021 ▸). Specifically for the samples here presented, we used a simplified version of CTF assuming a pure phase object, which was a valid approach for both samples as the total transmission is above 95%.

**Figure 5:**
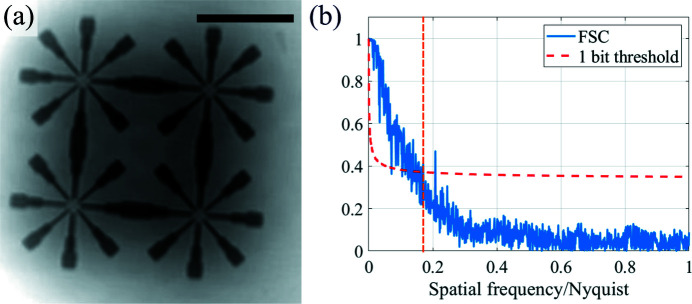
2D test pattern reconstructions (*a*) and resolution evaluation using Fourier ring correlation (*b*). The vertical orange-dashed line in (*b*) represents the focal-spot resolution limit. The scale bar corresponds to 4 µm, and the resolution estimated by the 1-bit threshold criterion is 79 nm.

**Figure 6:**
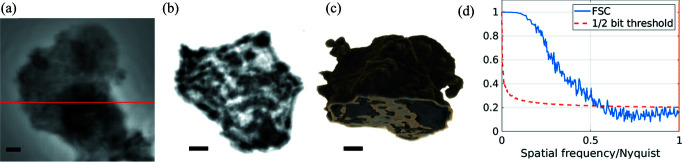
Chalk sample tomographic reconstruction. (*a*) Phase retrieved projection using pure-phase algorithms. (*b*) 3D reconstructed slice marked by the red line in (*a*). (*c*) 3D rendering of the chalk sample. (*d*) Fourier shell correlation results and resolution estimation, where the vertical orange-dashed line represents the focal-spot resolution limit. The scale bars correspond to 2 µm, and the resolution estimated by the half-bit threshold criterion is 155 nm.

Figure 5 ▸(*a*) depicts the phase reconstruction of the test pattern. Figure 6 ▸(*a*) shows one phase-retrieved projection of the chalk sample.

The chalk sample required extra steps for the 3D reconstruction. To accelerate the 3D reconstruction workflow, we rebinned the frames by a factor of three in both directions. This operation does not limit our resolution, given the expected resolution limit compared with the effective pixel size. Due to the mechanical instabilities of the tomographic setup, the rebinned projections had to be aligned. The alignment was performed using the joint re-projection algorithm (Gürsoy *et al.*, 2017 ▸). The final 3D reconstruction was obtained using the Gridrec algorithm together with Parzen filtering as implemented in *TomoPy* (Gürsoy *et al.*, 2014 ▸). Figures 6 ▸(*b*) and 6(*c*) show a slice of the chalk reconstruction and a visualization of the 3D volume, respectively.

### 3.2. Resolution evaluation

To evaluate the achieved resolution, we performed Fourier ring correlation (FRC) for the 2D test pattern and Fourier shell correlation (FSC) for the 3D test sample (Van Heel, 1987 ▸; van Heel & Schatz, 2005 ▸). For the 2D sample, we performed two independent reconstructions from two independent scans, each of them from 5 s acquisitions. The result of the FRC for the 2D sample is shown in Fig. 5 ▸(*b*). An apodization function with the FWHM of the focal spot (70 nm) was used to compute the FRC. One can see that the cut-off frequency over the Nyquist frequency is around 0.15, when the one-bit threshold criterion is applied (van Heel & Schatz, 2005 ▸). This frequency corresponds to a resolution of 79 nm, similar to the focal-spot size limit, which indicates the compatibility with diffraction-limited resolution. For the 3D sample, we performed two independent reconstructions by dividing the projection dataset into two, *i.e.* 650 projections evenly distributed for each 3D reconstruction. The results of the FSC, using the aforementioned apodization function and masking the pixels outside the reconstructed volume, are depicted in Fig. 6 ▸(*d*). From the FSC and a 45 nm voxel size, the estimated resolution was 155 nm using the 1/2 bit criterion.

## 4. Discussion

The here presented experiments demonstrate the unique capabilities of NanoMAX to explore length scales from the mesoscale down to the nanoscale by exploiting the unique focusing possibilities and high-coherent flux provided by MAX IV.

The 2D test pattern was reconstructed with diffraction-limited resolution. This was validated by using FRC as depicted in Fig. 5 ▸(*b*). The data were acquired with 5 s exposure for each frame. The chalk sample was exposed for 1 s per projection. This resulted in a 22 minute acquisition time per tomogram with 1300 projections and per distance. As we recorded tomograms at four distances, the total acquisition time was 88 minutes for this sample. The results presented here demonstrate the possibility to perform fast acquisitions and reconstructions at the mesoscale, exploiting the unique capabilities of NanoMAX.

Nonetheless, the current setup has certain limitations. First, the alignment of the sample to the center of rotation was done using manual stages together with an optical microscope. This is a time-consuming process, which can be improved by adding motorized linear stages on top of the rotation stage. Second, although the acquisition times were 1 s for the 2D and 3D samples, the total exposure time was much longer due to overheads. In fact, the tomographic scan took a total of 2 h. This unnecessary exposure of the sample to the X-rays while not acquiring data could be reduced by using a fast shutter. Third, to increase the sensitivity of the method while minimizing dose, one would like to exploit higher photon energies, which would also enable higher resolutions (Fourme *et al.*, 2012 ▸; Villanueva-Perez *et al.*, 2018 ▸). However, the expected detectable photons with the current setup at NanoMAX decreases at higher photon energies, as shown in Fig. 2 ▸. To improve the setup efficiency, one can think of using a detector with higher efficiency. On the other hand, there is a loss of efficiency at lower energies due to air absorption and scattering. This could be improved by minimizing the air path using a flight tube. Fourth, the setup would also benefit from cryogenic environments to image radiosensitive samples that can minimize or avoid the observed radiation damage.

## 5. Conclusions

We have presented the first experiments of in-line holography and holotomography at the NanoMAX beamline of MAX IV. We demonstrate that we can retrieve diffraction-limited images with short exposure times by exploiting the nano­focusing capabilities of NanoMAX and the coherent flux provided by MAX IV. This setup will constitute a tool to bridge microtomography and nanotomography capabilities by enabling the exploration of the mesoscale down to the nanoscale. In fact, this setup has the potential to acquire 3D tomographic information with resolutions around 100 nm with acquisition times of a few minutes for a single defocusing distance. This high spatial and temporal resolution can enable *in situ* and *operando* imaging experiments at NanoMAX. Finally, given the travel range of the sample along the optical axis that allows the acquisition of meaningful holograms and positioning the sample on the focal spot, we envision that in-line holography can be easily combined with scanning techniques to provide a good overview of the scanned region with equivalent resolution.
